# Research on mechanical properties of concrete by nano-TiC-BF-fly ash

**DOI:** 10.1038/s41598-024-55553-0

**Published:** 2024-02-27

**Authors:** Xin Yang, Kui Yu, Ke Li, Zhengjun Wang, Fengchun Ji, Mengyuan Li

**Affiliations:** 1 College of Water Resources and Electricity, Heilongjiang University, Harbin, 150080 China; 2 Key Laboratory of Impact and Structural Safety, Academy of Civil Engineering & Architecture, Nanyang Normal University, Nanyang, 473061 China; 3 Science and Technology on Advanced Ceramic Fibers and Composites Laboratory, College of Aerospace Science and Engineering, National University of Defense Technology, Changsha, 410073 People’s Republic of China

**Keywords:** Response surface methodology, Compound concrete, Mechanical properties, Deformation and damage characteristics, Mixing ratio, Micromorphology, Engineering, Materials science

## Abstract

Ultra-high-rise buildings require high concrete bearing capacity. Ordinary concrete often fails to meet the project requirements. Admixture of admixtures in concrete is a means of solution. Currently, studies on the incorporation of basalt fiber (BF) and fly ash (FA) in concrete are relatively mature. However, research on incorporating nano-Titanium Carbide (nano-TiC) in concrete is still relatively scarce, which has a lot of room for development. To further improve the mechanical properties of concrete, BF, and FA synergized with nano-TiC were incorporated into concrete to produce TBF concrete in this study. And Response Surface Methodology (RSM) was used to optimize the mechanical properties of concrete. The collapse and compressive deformation damage characteristics of concrete were analyzed. The microstructure of the cement matrix was analyzed by the SEM (Scanning Electron Microscope). An optimization model of the TBF concrete craving function was developed. Optimized ratios with compressive, split tensile, and flexural strengths as response objectives were obtained, and the accuracy of the optimized ratios was investigated using the same experimental conditions. The results of the study showed that FA increased the collapse of concrete, while nano-TiC and BF decreased the collapse of concrete. Under uniaxial compression, nano-TiC, FA, and BF together incorporated into concrete can improve its compressive damage state. Moderate amounts of nano-TiC, BF, and FA could improve the mechanical properties of concrete. Their optimal mixing ratio admixtures were 0.88%, 0.24%, and 5.49%, respectively. And the measured values under the same conditions were compared with the predicted values. The maximum difference in compressive strength was 6.09%. The maximum difference in split tensile strength was 7.14%. The maximum difference in flexural strength was 8.45%. This indicated that the accuracy of the RSM optimization model was good. A moderate amount of nano-TiC, FA, and BF could improve the densification of concrete.

## Introduction

Concrete, as an indispensable material in construction, played a key role in structural stability and bearing capacity^1,2^, and traditional cement-based concrete had limitations and deficiencies when faced with increasingly stringent environmental requirements^3,4^. Ordinary concrete had good compressive properties but poor tensile strength and strain characteristics, and its body was susceptible to cracking when subjected to bending stresses and tensile action^5^. Adding an appropriate amount of steel reinforcement in concrete to share the external force with the cement matrix could effectively reduce the cracks and improve the tensile stress of concrete^6^. This move, however, inevitably led to an increase in the cost of materials. Therefore, researchers used admixtures and added new materials in concrete to reduce the cost. It has been proved by numerous studies that adding fibers to concrete could effectively reduce cracks and improve the durability of concrete. The Basalt fiber (BF) was widely used in concrete due to its mechanical properties, lower cost, and resistance to alkaline corrosion^7^. Although mixing the appropriate amount of fibers in concrete could enhance its mechanical properties, it still could not meet the requirements of buildings with high-strength classes.

Normally, in order to improve the late strength and compactness of concrete, and to facilitate the pumping and transportation of concrete. Constructors often add the appropriate amount of fly ash (FA) to concrete. Therefore, FA has become a commonly used additive in concrete^8^. However, the amount of FA added in concrete was generally fixed and not necessarily the most accurate amount. To solve this problem, researchers have also adopted statistical methods to precisely find the appropriate amount of multiple admixtures in concrete. After a large number of engineering studies and applications, the mechanical properties of concrete could be improved by incorporating appropriate amounts of two or more admixtures into concrete. However, this also brings up three questions: first, could the multiple admixtures incorporated in the concrete achieve the desired effect? Second, how to determine the appropriate mix ratio of concrete? Third, could the mechanical properties of concrete be improved by the incorporation of other new materials?

Compound concrete had received attention from research researchers as a solution^9–11^. Sosa^12^ et al. mixed pyrophyllite, limestone, FA, and slag to obtain high-strength self-compacting concrete. Muhammad et al.^13^ incorporated nano-SiO_2_, alkaline activator, and sodium aluminate into concrete which improved the splitting tensile strength of concrete. Kumar et al.^14^ stated that the incorporation of FA, eggshell meal, and steel fibers could enhance the mechanical strength of concrete. Nirubha et al.^15^ improved the mechanical properties and durability of concrete by replacing part of the cement with dolomitic sand and nano-SiO_2_. Esmaeili et al.^16^ incorporated lightweight bentonite and steel fibers into concrete, improved the cracking resistance, and delayed the cracking time of concrete. Naran et al.^17^ used waste plastics, FA, and recycled glass powder as additives to concrete. The concrete was tested for hardening properties and analyzed microscopically, and a denser concrete was obtained.

The mechanical properties of concrete could be enhanced by incorporating an appropriate amount of admixtures in concrete^12–20^, but it also faces the problem of material proportioning. Researchers usually used a control group to find the additive ratio of concrete. But, it would result in an excessive number of experimental groups and an imprecise amount of additives. For this reason, researchers then resorted to Response Surface Optimization (RSM) to solve this problem. The RSM could provide an in-depth study of the behavior of concrete under different factors and their levels through statistical modeling and analysis^21^. Currently, the RSM has been widely used to solve the problem of optimizing the proportion of composite materials in the field of concrete^22–25^. Norozi et al.^26^ incorporated limestone, steel slag, and copper slag into concrete. The RSM was used to optimize the concrete mix ratio and improve the resistance of concrete pavement to cracking. Adamu et al.^27^ incorporated powdered activated carbon (PAC) into concrete to address the characteristics of date palm fiber (DPF) in reducing the compressive strength of concrete. And the RSM was used to optimize DPF and PAC as variables. It was pointed out that an appropriate amount of DPF and PAC can enhance the strength of concrete. Zamir et al.^28^ substituted marble waste and stone dust for coarse and fine aggregates of concrete respectively. Statistical modeling by the RSM improved the durability of concrete. Song et al.^29^ optimized the production process of soybean residue protein (SRP) by the RSM and incorporated it into concrete which improved the mechanical properties of concrete. Murali et al.^30^ substituted rubber crumb from waste tires for some of the fine aggregates and fly ash for some of the cement. Optimization of compressive and splitting tensile strength of concrete by the RSM and high-strength concrete was obtained. Hau et al.^31^ used crumb rubber and graphene oxide to replace part of the cement. The mix ratio of concrete was optimized by the RSM, which improved the deformation characteristics and increased the mechanical strength. Therefore, the amount of concrete additives is optimized by the RSM. This initiative, not only reduces the number of experimental groups, but also obtains more precise amounts of substance^26–34^.

**Figure 1:**
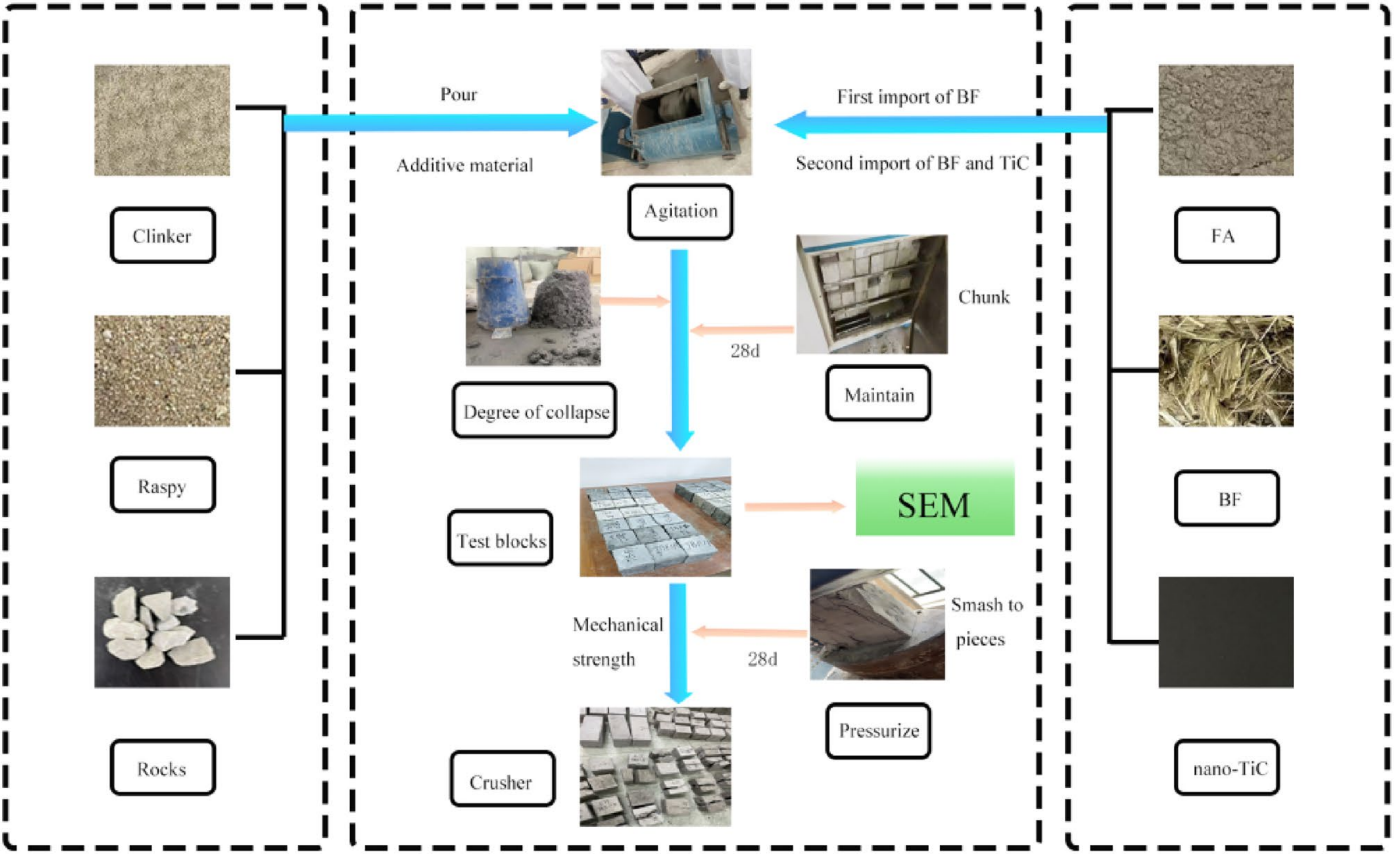
Flowchart of TBF production.

Currently, there were relatively few studies on the incorporation of nano-Titanium Carbide (nano-TiC) into concrete, which has a great deal of room for development. Nano-TiC is a nanomaterial with a large specific surface area, high surface activity, and high-temperature resistance, which has been widely used in ceramics fabrication and aerospace applications^35^. Based on previous studies, an appropriate amount of nanomaterials incorporated into concrete could improve its mechanical properties^36–38^. So could the incorporation of nano-TiC into concrete also have the effect of enhancing the mechanical strength of concrete? On the other hand, BF and FA blended into concrete concretes are relatively well established. To further improve the mechanical properties of concrete, BF, and FA synergized with nano-TiC were incorporated into concrete for research in this paper. Optimization experiments were carried out using the RSM-BBD (RSM-Box-Behnken) with the admixture of nano-TiC, BF, and FA as the response factors and the compressive, split tensile, and flexural strengths of concrete as the response values. The response surface optimization model and thirst function were established to analyze the influence law of each factor. The pressurized state of concrete was analyzed. The SEM(Scanning Electron Microscope) was used to observe the morphology of concrete after crushing. Providing the theoretical value and reference basis for researchers to study high-strength concrete (Fig. 1).

## Materials and methods

### Materials

Ordinary silicate cement P.O 42.5 was the main cementitious material for this experiment. Water reducing rate ≥ 25% of the polyhydroxy acid system of highly efficient water reducing agent. Medium sand with a fineness modulus of 2.76 was used as fine aggregate. Crushed stone with a continuous particle size of 13 ~ 37 mm was used as coarse aggregate. The FA used in the experiment was secondary fly ash, and the nano-TiC was from Top Metal Materials Co. The FA and nano-TiC replaced part of the cement. Laboratory tap water was used as the mixing water for concrete.BF (physical and mechanical properties are shown in Table 1) was from Sichuan TuoXin Basalt Industry Co.Ltd.BF was repeatedly rinsed with Wahaha pure water before being added to the concrete and put into a constant temperature blast drying oven and heated and dried at 110°C for 2h, and then allowed to dry completely after resting.

**Table 1:** Physical and mechanical properties of BF.

Length/mm	Monofilament diameter/µm	Density/g cm^−3^	Elastic modulus/GPa	Tensile strength/MPa	Elongation at break/%
12	13	2.65 ~ 2.70	95 ~ 115	3300 ~ 4500	2.4 ~ 3.0

### Experimental design theory and concrete fabrication methods

#### The response surface method design theory

The Response surface methodology was a statistical method commonly used to optimize experimental design and find parameters. It described the relationship between the independent and dependent variables by building a mathematical model and used this model to seek the optimal experimental conditions or optimization scheme. In experimental design, the response surface method used maximizing or minimizing the dependent variable as the response objective which determined the response value of the dependent variable. It would help researchers to conduct experiments more efficiently under limited experimental conditions and find the optimal experimental parameters. The earliest RSM was proposed by Box and Wilson et al. with the whole set of experimental principles in 1951. After that, the RSM has been widely used in experimental design simulation^39,40^.

Since the relationship between the response values and the free factors is unknown, the first step in RSM is to determine the fitted relationship between the response values and the free factors. The function used for this relationship is an infinite polynomial of low order. Usually, the function is mostly a quadratic polynomial, i.e., the functional equation is shown in Eq. (1):

1 $${y}_{0}={a}_{0}+\sum_{i=1}^{k}{a}_{i}{x}_{i}+\sum_{i=1}^{k}{a}_{ii}{{x}_{i}}^{2}+\sum_{i=1}^{k}\sum_{i=k}^{k}{a}_{ij}{x}_{i}{x}_{j} i<j$$

The $k$ in the formula is the number of variables. When the independent variables are two, the formula for the response surface is defined as (2):

2 $${y}_{0}={a}_{0}+{a}_{1}{x}_{1}+{a}_{2}{x}_{2}+{a}_{3}{{x}_{1}}^{2}+{a}_{4}{{x}_{2}}^{2}+{a}_{5}{x}_{1}{x}_{2}$$

When ${x}_{3}={{x}_{1}}^{2}$, ${x}_{4}={{x}_{2}}^{2}$, ${x}_{5}={x}_{1}{x}_{2}$. Equation (3) is then obtained after the variation of Eq. (2):

3 $${y}_{0}={a}_{0}+{a}_{1}{x}_{1}+{a}_{2}{x}_{2}+{a}_{3}{x}_{3}+{a}_{4}{x}_{4}+{a}_{5}{x}_{5}$$

When the total number of experiments is n. The formulas for the response surface are shown in Eqs. (4) and (5):

4 $$\widetilde{Y}=\widetilde{X}\widetilde{A}+\widetilde{e}$$

5 $$\widetilde{Y}=\left[\begin{array}{c}{y}_{1}\\ {y}_{2}\\ \begin{array}{c}.\\ \begin{array}{c}.\\ \begin{array}{c}.\\ {y}_{n}\end{array}\end{array}\end{array}\end{array}\right]\widetilde{X}=\left[\begin{array}{c}1\\ 1\\ \begin{array}{c}.\\ \begin{array}{c}.\\ \begin{array}{c}.\\ 1\end{array}\end{array}\end{array}\end{array}\begin{array}{c}{x}_{11}\\ {x}_{21}\\ \begin{array}{c}.\\ \begin{array}{c}.\\ \begin{array}{c}.\\ {x}_{n1}\end{array}\end{array}\end{array}\end{array}\begin{array}{c}{x}_{12}\\ {x}_{22}\\ \begin{array}{c}.\\ \begin{array}{c}.\\ \begin{array}{c}.\\ {x}_{n2}\end{array}\end{array}\end{array}\end{array}\begin{array}{c}.\\ .\\ \begin{array}{c}.\\ \begin{array}{c}.\\ \begin{array}{c}.\\ .\end{array}\end{array}\end{array}\end{array}\begin{array}{c}.\\ .\\ \begin{array}{c}.\\ \begin{array}{c}.\\ \begin{array}{c}.\\ .\end{array}\end{array}\end{array}\end{array}\begin{array}{c}{x}_{1k}\\ {x}_{2k}\\ \begin{array}{c}.\\ \begin{array}{c}.\\ \begin{array}{c}.\\ {x}_{nk}\end{array}\end{array}\end{array}\end{array}\right]\widetilde{A}=\left[\begin{array}{c}{a}_{0}\\ {a}_{1}\\ \begin{array}{c}.\\ \begin{array}{c}.\\ \begin{array}{c}.\\ {a}_{n}\end{array}\end{array}\end{array}\end{array}\right]\widetilde{e}=\left[\begin{array}{c}{e}_{1}\\ {e}_{2}\\ \begin{array}{c}.\\ \begin{array}{c}.\\ \begin{array}{c}.\\ {e}_{n}\end{array}\end{array}\end{array}\end{array}\right]$$

where $\widetilde{e}$ is the error vector and the parameter vector $\widetilde{A}$ comes from the predictor $\widetilde{b}$. And $\widetilde{b}$ obeys Eq. (6):

6 $$\widetilde{b}={\left({\widetilde{X}}^{T}X\right)}^{-1}{\widetilde{X}}^{T}\widetilde{Y}$$

And the variance–covariance of $\widetilde{b}$ obeys Eq. (7):

7 $${\text{cov}}({b}_{i},{b}_{j})={C}_{ij}={s}^{2}{\left({\widetilde{X}}^{T}X\right)}^{-1}$$

where $s$ is the error of $\widetilde{Y}$ whose predicted value obeys Eq. (8).

8 $${s}^{2}=\frac{S{S}_{E}}{n-k-1}$$

$S{S}_{E}$ is the residual sum of squares and obeys Eq. (9):

9 $$S{S}_{E}={\widetilde{Y}}^{T}\widetilde{Y}-{\widetilde{b}}^{T}{\widetilde{X}}^{T}{\widetilde{X}Y}^{T}$$

To improve the prediction accuracy of the response surface method, the multiple adjusted ${{R}^{2}}_{adj}$ obeys Eq. (10).

10 $${{R}^{2}}_{adj}=1-\frac{S{S}_{E}/(n-k-1)}{{S}_{yy}/(n-1)}$$

The ${S}_{yy}$ is the total sum of squares and obeys Eq. (11).

11 $${S}_{yy}={\widetilde{Y}}^{T}\widetilde{Y}-\frac{{({\sum }_{i=1}^{n}{y}^{i})}^{2}}{n}$$

where ${{R}^{2}}_{adj}\in \left[\mathrm{0,1}\right]$, as ${{R}^{2}}_{adj}$ gets closer to 1, it indicates a higher response surface accuracy. The results of the response surface obey Eq. (12):

12 $${t}_{0}=\frac{{b}_{j}}{\sqrt{{S}^{2}{C}_{jj}}}$$

where ${C}_{jj}$ comes from Eq. (7). The ${t}_{0}$ is the statistic and ${b}_{j}$ is the coefficient. If ${t}_{0}$ satisfies $\left|{t}_{0}\right|>{t}_{a/2,n-k-1}$. The null hypothesis ${\beta }_{j}=0$ is rejected. where $\alpha$ is the acceptable probability of error type I occurring and $\beta$ is the probability of error type II occurring.

#### The optimization principle of the thirst function method

The desirability function method is also called the satisfaction function method.It first converts individual response quantities into individual thirst values on the interval [0,1] and then finds the weighted geometric mean of all individual thirst values to maximize it. Thus, the multi-response problem is transformed into a single-response problem. The method is a simple, easy, and widely used optimization method for multi-response outputs.

Define a single thirst value ${d}_{i}$, ${d}_{i}\in [\mathrm{0,1}]$, corresponding to the response value. The closer the response value is to the target, the closer its thirst value is to 1. When the response value exceeds the specification or is close to the specification, its thirst value is close to 0 or equal to 0. The formulas for the Lookout Large and Lookout Small satisfaction algorithms are shown below. The ${L}_{i}$, ${U}_{i}$, and ${T}_{i}$ are the previous, next, and target values for the $i$th response, respectively. The index r is a row-tuning function, reflecting how much the goal is satisfied. The thirst function D is the power exponential product of the thirst values of each response, see Eq. (15). where ${\omega }_{i}$ is the weight of the $i$th response satisfying ${\omega }_{i}\in [\mathrm{0,1}]$ and $\sum {\omega }_{i}=1$.

13 $${d}_{i}({Y}_{i}{)}_{max}=\left\{\begin{array}{ll}0, & {Y}_{i}<{L}_{i}\\ \left(\frac{{Y}_{i}-{L}_{i}}{{T}_{i}-{L}_{i}}\right){r}_{i}, & {L}_{i}\le {Y}_{i}\le {T}_{i}\\ 1, & {Y}_{i}>{L}_{i}\end{array}\right.$$

14 $${d}_{i}({Y}_{i}{)}_{max}=\left\{\begin{array}{ll}0, & {Y}_{i}<{T}_{i}\\ \left(\frac{{U}_{i}-{Y}_{i}}{{U}_{i}-{T}_{i}}\right){r}_{i}, & {T}_{i}\le {Y}_{i}\le {U}_{i}\\ 1, & {Y}_{i}>{U}_{i}\end{array}\right.$$

15 $$D={\left[{\coprod }_{i}({d}_{i}({y}_{i})){\omega }_{i}\right]}^{\frac{1}{\sum {\omega }_{i}}}$$

#### The preparation of TBF concrete

Based on the results of the indoor tests, the final mix ratio of the concrete was determined. In this case, the cement was 420 kg m^−3^. The sand rate was 38%. The water-cement ratio was 0.5, and the amount of water-reducing agent was 0.62%. Three factors, nano-TiC, BF, and FA, were selected for the experiment, denoted by *A*, *B*, and *C*, respectively. In this case, the reducing agent, nano-TiC, and FA are the percentage of cement mass and BF is the volumetric admixture. Response values were chosen as concrete compressive, split tensile, and flexural strengths. The Box-Behnken design three-factor, a three-level scheme in Design-Expert12 was used. The factors and levels of each test are shown in Table 2, and the test results are presented in Table 3.

**Table 2:** TBF concrete response factors and response volume design table.

Brochure	Unit	Typology	Level		
			− 1	0	1
*A:* nano-TiC	%	Factor	0	1.25	2.5
*B:* BF	%	Factor	0	0.15	0.3
*C:* FA	%	Factor	0	7.5	15
Compressive strength	MPa	Response value			
Splitting tensile strength	MPa	Response value			
Flexural strength	MPa	Response value

**Table 3:** Results of experimental design.

Test group	A	B	C	Degree of collapse	Compressive strength	Splitting tensile strength	Flexural strength	Modulus of elasticity
	%	%	%	mm	MPa	MPa	MPa	GPa
TBF0	0	0	0	121	37.5	4.5	6.89	28.6
TBF1	0	0.15	15	102	38.2	4.4	6.62	31.4
TBF2	1.25	0.15	7.5	92	48.5	4.8	7.85	39.1
TBF3	0	0.15	0	86	42.2	4.6	7.01	32.3
TBF4	2.5	0.15	0	75	43.4	4.4	6.73	36.1
TBF5	1.25	0.15	7.5	90	46.9	4.9	7.84	39.2
TBF6	1.25	0.3	0	80	38.0	5.4	8.07	29.4
TBF7	0	0.3	7.5	90	38.1	5.6	8.25	30.3
TBF8	1.25	0	0	115	40.8	4.2	6.41	37.6
TBF9	1.25	0.15	7.5	87	48.4	4.7	7.92	39.3
TBF10	1.25	0.15	7.5	96	47.7	4.8	7.81	38.7
TBF11	2.5	0.3	7.5	62	33.8	5.5	8.22	31.2
TBF12	1.25	0.3	15	85	32.5	4.8	7.56	26.1
TBF13	1.25	0	15	151	36.1	4.1	6.32	29.9
TBF14	1.25	0.15	7.5	97	49.5	4.9	8.01	38.9
TBF15	2.5	0	7.5	122	43.4	4.6	6.91	35.2
TBF16	0	0	7.5	136	35.5	4.5	6.81	29.4
TBF17	2.5	0.15	15	82	39.1	4.3	6.59	31.9

The cement, sand, and gravel aggregate were weighed and poured into the concrete single-shaft mixer for 3 min. and then nano-TiC, FA, and BF were added and mixed for 5 min. The polyhydroxy acid highly efficient water-reducing agent was added and weighed and the appropriate amount of water was put into the mixer and mixed for 2 min. And then the concrete was poured into the molds and manually vibrated, to produce the TBF-type concrete. And test the collapse of concrete. Compressive and split tensile strength tests were performed for the cube specimens (100 × 100 × 100 mm). Flexural strength tests were performed for the prismatic specimens (100 × 100 × 400 mm). Concrete mechanical properties of experiments refer to the "Standard for test methods of concrete physical and mechanical properties" (GB/T 50081-2019). The experiment was tested in groups. Each group of test blocks was 3 and the measurements on day 28 were recorded.

## Results and discussion

### Analysis of the concrete collapse

**Figure 2:**
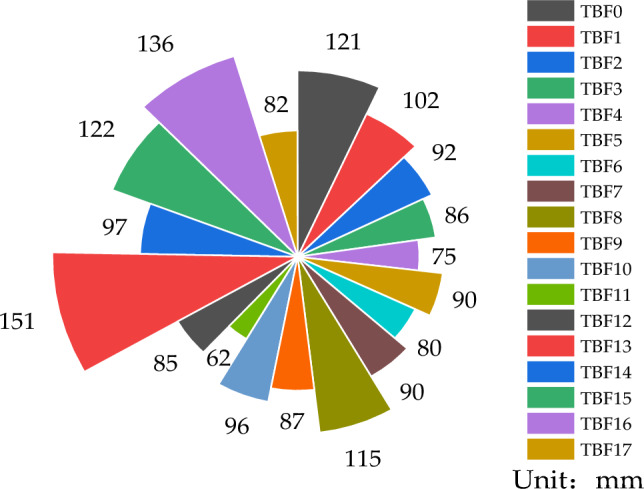
Concrete collapse.

The collapse of concrete is shown in Fig. 2, which reflects the effect of nano-TiC, BF, and FA on the collapse of concrete. When the FA dosage was certain, the collapse of concrete decreased with the increase of BF dosage. For example, for concrete test blocks TBF7 and TBF16, the FA dosing was 7.5%, the volume dosing of BF was increased from 0% to 0.3%, and the concrete caving degree decreased from 136 to 90 mm. Analyzed from the benchmark blocks, such as TBF0 and TBF3, the volume dosing of BF was increased from 0 to 0.15%, and the concrete caving degree decreased from 121 to 86 mm. The reason was mainly that BF has certain ductility and adhesion, which can form a mesh structure in concrete and inhibit the flow of aggregates, thus reducing the collapse of concrete^41^.

When FA was certain, the admixture of nano-TiC in concrete decreased the collapse of concrete. For example, in TBF15 and TBF16, the FA admixture was 7.5%, the admixture of nano-TiC was increased from 0 to 2.5%, and the concrete's caving degree decreased from 136 to 122 mm. Analyzed from the benchmark blocks, such as TBF0 and TBF8, the concrete's caving degree decreased from 121 to 115 mm. The reason for this may be because the large comparative area of nano-TiC requires more water to reduce the viscosity of the cement paste.

When the nano-TiC was certain, the collapse of concrete was elevated with the increase of FA. For example, in TBF8 and TBF13, when the nano TiC dosage is 1.25%, the FA dosage increases from 0 to 15% and the caving capacity increases from 115 to 151 mm. Analyzed from the benchmark block, such as in TBF0 and TBF16, the caving capacity of the concrete increases from 121 to 136 mm. The main reason for this is that the interfacial friction of the mortar aggregates decreases due to fly ash due to the morphological effect of fly ash, which improved the fluidity of concrete collapse^42^. In conclusion, moderate amounts of nano-TiC and FA can improve the concrete collapse, while the concrete collapse shows a decreasing trend due to the increase of BF admixture.

### The concrete failure modes

**Figure 3:**
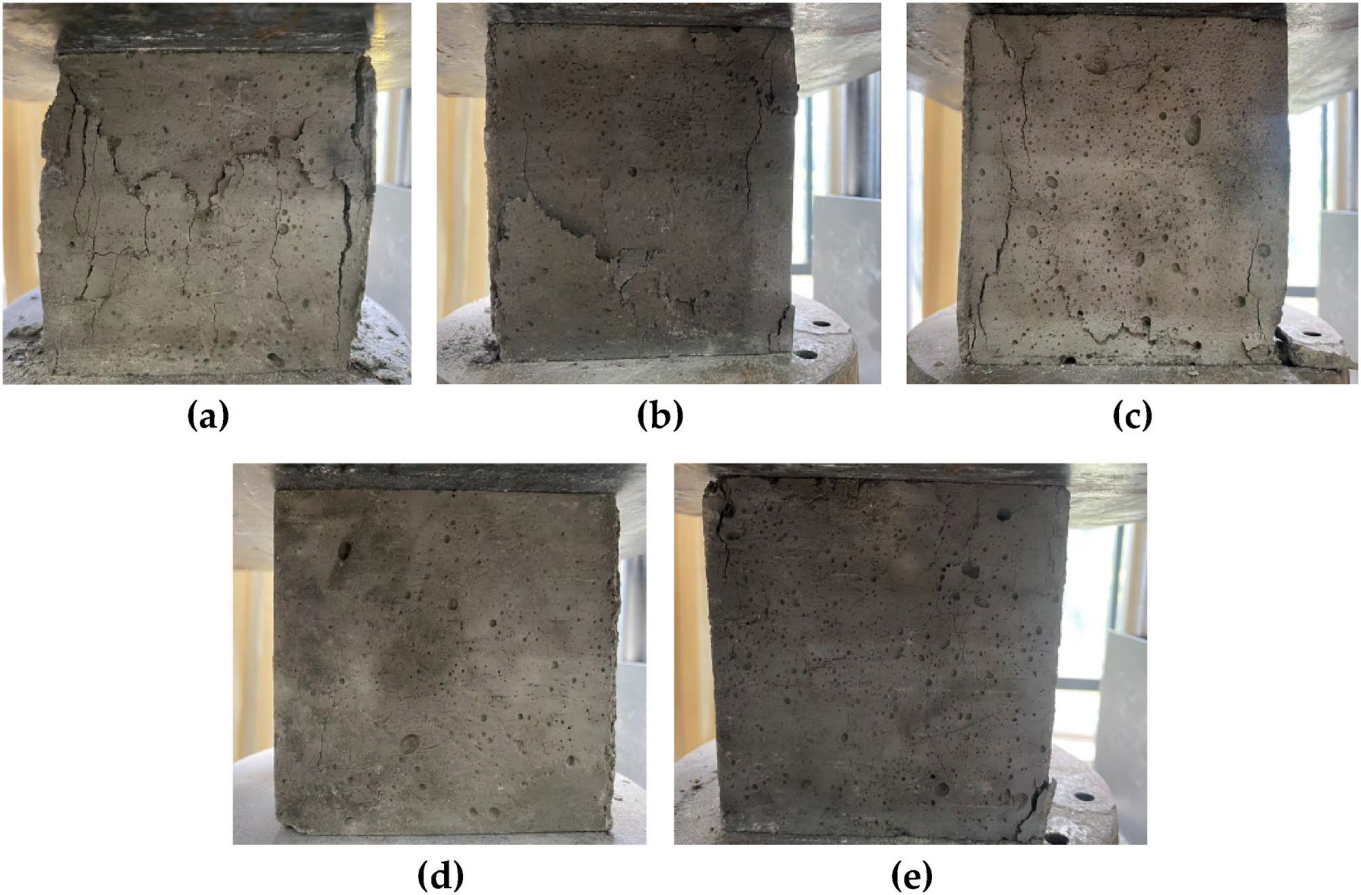
Concrete uniaxial compression damage pattern: (**a**) TBF0, (**b**) TBF8, (**c**) TBF16, (**d**) TBF3, (**e**) TBF5.

The internal admixture materials of concrete play an important role in the performance and durability of building structures. If the concrete mix ratio and raw materials are not used properly, it may lead to problems such as insufficient concrete strength and cracking, which ultimately lead to structural failure. To further evaluate the effect of nano-TiC, BF, and FA on the compressive damage of concrete. The failure modes of plain concrete TBF0, single-doped nano-TiC (TBF8), single-doped FA (TBF16), single-doped BF (TBF3), and mixed-doped nano-TiC, BF, and FA (TBF5) on concrete under uniaxial-compression were investigated. The specific failure modes are shown in Fig. 3.

The compressive failures of TBF0, TBF8, TBF16, TBF3, and TBF5 are shown in Fig. 3. The cracks appeared in TBF0, TBF8, and TBF16 during the pressurization process from the site of stress concentration, and with the increasing pressure, the cracks continued to expand and penetrate the test block. The aggregate on the cement surface was dislodged and the concrete could not maintain its integrity. The TBF3 and TBF5 had only a few cracks produced after reaching the destructive load. The surface of some specimens had no or little shedding, and the specimens were able to maintain their integrity better. The addition of the BF in concrete could better maintain the integrity of the specimen and improve the damaged state of the specimen. The results were consistent with the findings of Chen^43^ et al.

### Mechanical strength of modified concrete

#### RSM-BBD intensity prediction model

A quadratic polynomial regression equation for this model was established by nonlinearly fitting the results of the response surface experiment (Table 3) according to RSM-BBD. The relationship between the response predictions and the independent variable impact factor is as follows:

16 $$f_{cu} = 48.20 + 0.71\rho_{t} - 1.67\rho_{b} - 2.31\rho_{f} - 3.05\rho_{t} \rho_{b} - 0.075\rho_{t} \rho_{f} - 0.20\rho_{b} \rho_{f} - 3.31\rho_{t}^{2} - 7.19\rho_{b}^{2} - 4.16\rho_{f}^{2}$$

17 $$f_{t} = 4.82 - 0.037\rho_{t} + 0.49\rho_{b} - 0.13\rho_{f} - 0.050\rho_{t} \rho_{b} + 0.025\rho_{t} \rho_{f} - 0.13\rho_{b} \rho_{f} + 0.015\rho_{t}^{2} + 0.21\rho_{b}^{2} - 0.41\rho_{f}^{2}$$

18 $$f_{f} = 7.89 - 0.030\rho_{t} + 0.71\rho_{b} - 0.14\rho_{f} - 0.032\rho_{t} \rho_{b} + 0.062\rho_{t} \rho_{f} - 0.11\rho_{b} \rho_{f} - 0.35\rho_{t}^{2} + 7.000E - 003\rho_{b}^{2} - 0.80\rho_{f}^{2}$$

where, ${f}_{cu}$ is the compressive strength of concrete. ${f}_{t}$ is the splitting tensile strength of concrete. ${f}_{f}$ is the flexural strength of concrete. ${\rho }_{t}$ is the dosage of nano-TiC. ${\rho }_{b}$ is the volumetric dosage of BF. ${\rho }_{f}$ is the dosage of FA.

#### RSM model test

As can be seen from Tables 4, 5 and 6, the P-value of each model is less than 0.01. This indicated that the models were highly accurate and statistically significant. The p-values for the misfit terms were all greater than 0.05, indicating a good model fit. The model-corrected coefficients of determination were 0.9785, 0.9545, and 0.9859, indicating that the model explained 97.85%, 95.45%, and 98.59% of the variation in response values, respectively. The model prediction coefficients of determination were 0.9620, 0.8145, and 0.9497, respectively. the model coefficients of variation (C.V.) were less than 10%, and the sound-to-noise ratios were greater than 4. This indicates that the models have a high degree of fit and that the above models could be used to continue with the optimization of the subsequent analyses.

**Table 4:** ANOVA of the compressive strength regression model.

Source	Sum of square	Degree of freedom	Mean square	*F* value	*P* value	
Model	477.10	9	53.01	81.78	< 0.0001	Significant
A: Nano-TiC	4.06	1	4.06	6.27	0.0408	
B: BF	22.44	1	22.44	34.63	0.0006	
C: FA	42.78	1	42.78	66.00	< 0.0001	
AB	37.21	1	37.21	57.40	0.0001	
AC	0.022	1	0.022	0.035	0.8575	
BC	0.16	1	0.16	0.25	0.6345	
A^2^	46.20	1	46.20	71.27	< 0.0001	
B^2^	217.52	1	217.52	335.56	< 0.0001	
C^2^	72.95	1	72.95	112.55	< 0.0001	
Residual	4.54	7	0.65			
Lack of fit	0.78	3	0.26	0.28	0.8409	Not significant
Cor total	481.64	16				

Adj R-Squared = 0.9785, Pred R-Squared = 0.9620, C.V. = 1.95%, Adequate precision = 25.162.

**Table 5:** Analysis of variance of cleavage tensile strength regression model.

Source	Sum of square	Degree of freedom	Mean square	*F* value	*P* value	
Model	2.98	9	0.33	38.29	< 0.0001	Significant
A: nano-TiC	0.011	1	0.011	1.30	0.2914	
B: BF	1.90	1	1.90	219.98	< 0.0001	
C: FA	0.13	1	0.13	14.46	0.0067	
AB	0.010	1	0.010	1.16	0.3178	
AC	2.500E−003	1	2.500E−003	0.29	0.6074	
BC	0.063	1	0.063	7.23	0.0311	
A^2^	9474E−004	1	9474E−004	0.11	0.7503	
B^2^	0.19	1	0.19	22.52	0.0021	
C^2^	0.71	1	0.71	81.89	< 0.0001	
Residual	0.061	7	8.643E−003			
Lack of fit	0.033	3	0.011	1.55	0.3329	Not significant
Cor total	3.04	16				

Adj R-Squared = 0.9545, Pred R-Squared = 0.8145, C.V. = 1.96%, Adequate Precision = 20.862.

**Table 6:** Analysis of variance of the regression model for flexural strength.

Source	Sum of square	Degree of freedom	Mean square	*F* value	*P* value	
Model	7.58	9	0.84	124.99	< 0.0001	Significant
A: nano-TiC	7.200E−003	1	7.200E−003	1.07	0.3358	
B: BF	3.99	1	3.99	591.85	< 0.0001	
C: FA	0.16	1	0.16	23.67	0.0018	
AB	4.225E−003	1	4.225E−003	0.63	0.4546	
AC	0.016	1	0.016	2.32	0.1717	
BC	0.044	1	0.044	6.54	0.0377	
A^2^	0.50	1	0.50	74.55	< 0.0001	
B^2^	2.063E−004	1	2.063E−004	0.031	0.8661	
C^2^	2.71	1	2.71	402.69	< 0.0001	
Residual	0.047	7	6.742E−003			
Lack of fit	0.021	3	7.158E−003	1.11	0.4421	Not significant
Cor total	7.63	16				

Adj R-Squared = 0.9859, Pred R-Squared = 0.9497, C.V. = 1.12%, Adequate Precision = 31.262.

From ANOVA shows. The effect of nano-TiC on the compressive strength of concrete is significant and the effect of BF and FA on the compressive strength of concrete is highly significant. the effect of BF and FA on the splitting tensile and flexural strength of concrete is highly significant. From the interaction, the interaction of nano-TiC and BF has a highly significant effect on the compressive strength of concrete. The interaction of FA and BF has a significant effect on the splitting tensile and flexural strength of concrete.

#### Compressive strength of modified concrete

**Figure 4:**
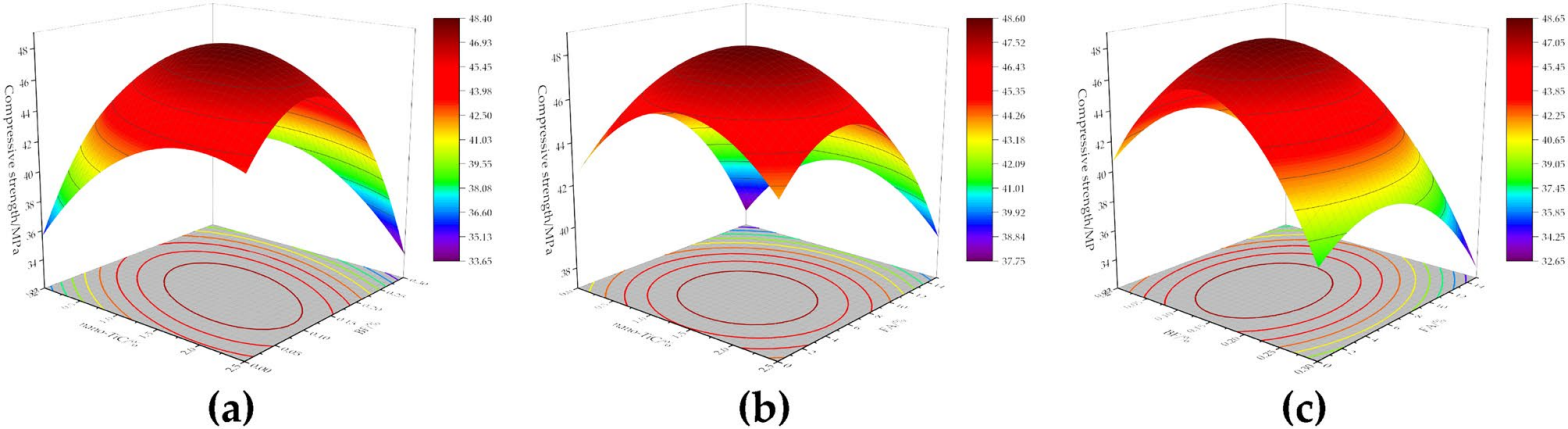
Compressive response surface diagram of concrete: (**a**) nano-TiC and BF, (**b**) nano-TiC and FA, (**c**) BF and FA.

Figure 4 shows the response surface plot of the effect of the interaction of factors on the 28-day compressive strength of concrete. From Fig. 4a, the compressive strength first increases and then decreases with the increase of nano-TiC and BF admixture. The compressive strength of concrete was higher when the nano-TiC dosage was located in the range of 1.0% to 2.0% and the volume dosage of BF was located in the range of 0.075% to 0.15%. From Fig. 4b, the compressive strength of concrete increased first and then decreased with the increase of nano-TiC and FA dosage. The compressive strength of concrete was higher when the dosage of nano-TiC was located in the range of 1.0% to 2.0% and the dosage of FA was located in the range of 2% to 7%. From Fig. 4c, the compressive strength first increased and then decreased with the increase of BF and FA admixture. The compressive strength value of concrete was higher when the volume admixture of BF was located at 0.1% to 0.2% and the admixture of FA was located at 2% to 8%.

From the above results, the addition of the appropriate amount of nano-TiC, BF, and FA to concrete could improve the compressive properties of concrete. The main reason for the increase in the compressive strength of concrete was that the appropriate amount of FA incorporated into concrete had an "aggregate effect" and reduced the interfacial friction between aggregate particles^44^. The nano-TiC, BF, and FA work together to improve the compactness of the concrete, which has a certain "filling" effect and reduces the defects inside the concrete, and the three-dimensional mesh structure formed by the BF in the concrete increases the resistance to deformation of the concrete^7^. However, excessive amounts of nano-TiC, FA, and BF could cause "agglomeration" in the cement matrix. This reduced the bonding properties between the aggregate and the matrix, thus reducing the compressive capacity of the concrete. The optimum mixing ratio for compressive strength was 1.48% for nano-TiC, 0.13% for BF, and 5.44% for FA.

#### The splitting tensile strength of modified concrete

**Figure 5:**
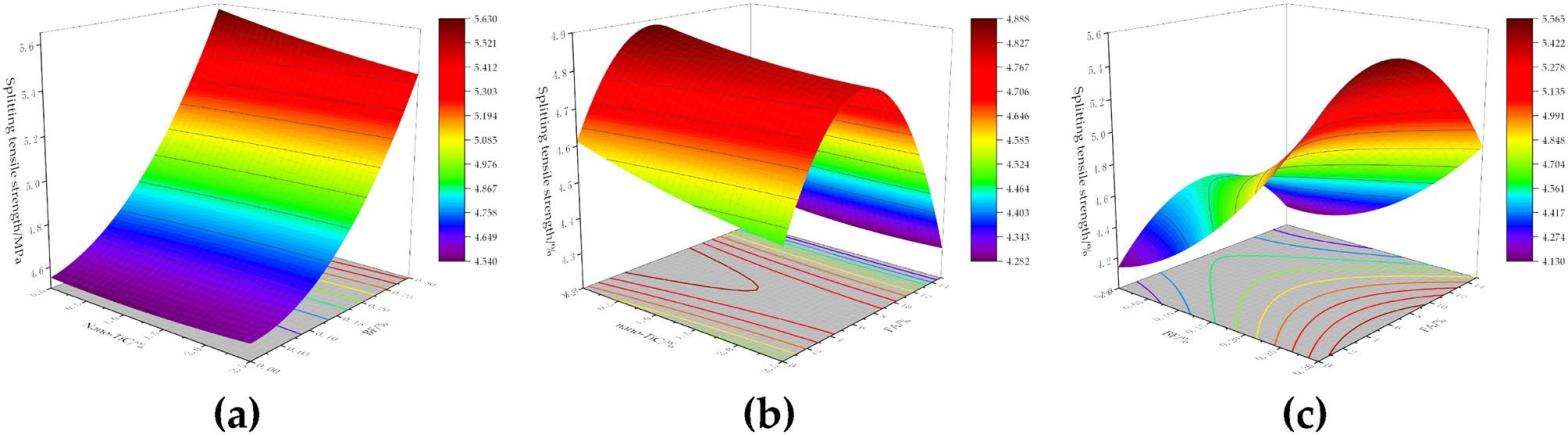
Surface plot of concrete splitting tensile response: (**a**) nano-TiC and BF, (**b**) nano-TiC and FA, (**c**) BF and FA.

Figure 5 shows the response surface plot of the effect of the interaction of factors on the 28-day splitting tensile strength of concrete. From Fig. 5a, the splitting tensile strength of concrete increased continuously with the increase in the volume admixture of BF when the admixture of nano-TiC was certain. The splitting tensile strength of concrete was enhanced by 22.22% when the volume admixture of BF was increased from 0% to 0.3% and the admixture of nano-TiC was increased from 0 to 2.5%. From Fig. 5b, the splitting tensile strength of concrete increased firstly and then decreased with the increase of FA dosage when the dosage of nano-TiC was certain. The splitting tensile strength of concrete was higher when the dosage of nano-TiC was located at 0% to 1.5% and the dosage of FA was located at 4% to 8%. From Fig. 5c, the splitting tensile strength of concrete was higher when the dosage of BF was located at 0.275% ~ 0.3% and the dosage of FA was located at 1% ~ 9%.

From the above results, the addition of BF to concrete helped to increase the splitting tensile strength of concrete. This was mainly because BF bears part of the tensile force at the section, which reduced the stress concentration at the concrete cracks and increased the ultimate tensile strain of the concrete^45^. The reactive substances such as silicates in FA reacted with the hydration product Ca(OH)_2_ in the cement to form a gel matrix that improved the splitting tensile capacity of the concrete^46^. The synergistic incorporation of nano-TiC further compensated the internal defects of concrete. The optimum mix ratios for splitting tensile strength were 0.42% for nano-TiC, 0.30% by volume for BF, and 4.67% for FA.

#### The flexural strength of modified concrete

**Figure 6:**
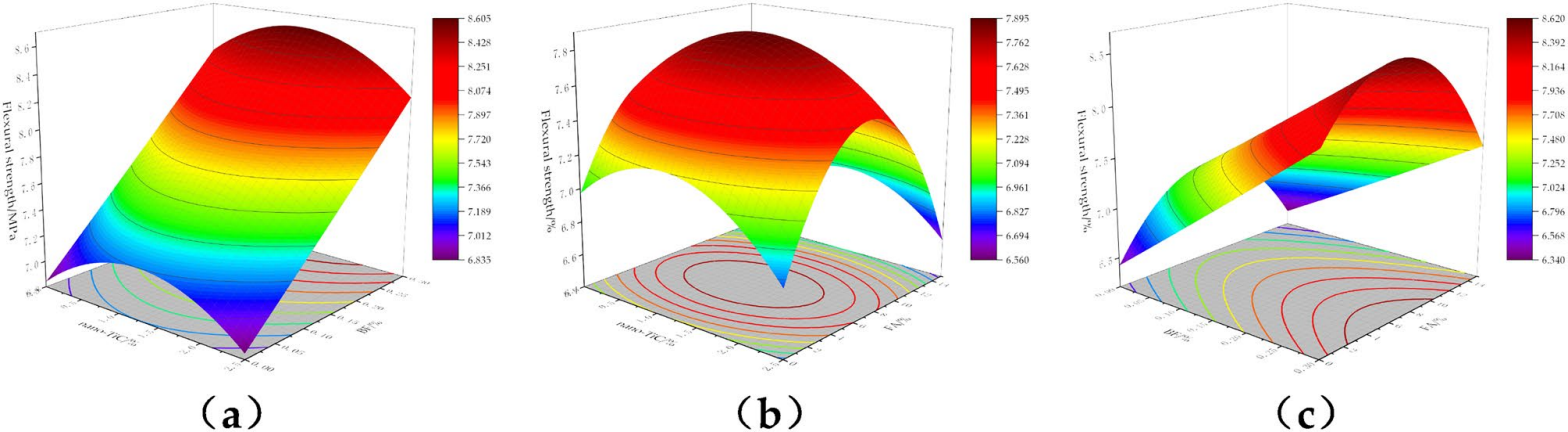
Corresponding surface plots of flexural strength of concrete: (**a**) nano-TiC and BF, (**b**) nano-TiC and FA, (**c**) BF and FA.

Figure 6 shows the response surface plot of the effect of the interaction of factors on the 28-day flexural strength of concrete. From Fig. 6a, when the dosage of nano-TiC was certain, the flexural strength of concrete increased gradually with the increase of the volume dosage of BF. When the dosage of nano-TiC is 0% and the volume dosage of BF was increased from 0 to 0.3%, the flexural strength of concrete was enhanced by 21.14%. From Fig. 6b, the flexural strength shows a trend of increasing and then decreasing with the increase of nano-TiC and FA admixture. The flexural strength of concrete was higher when the dosage of nano-TiC was located in the range of 0.75% to 2.0% and the dosage of FA was located in the range of 4% to 8%. From Fig. 6c, when the dosage of FA was certain, the flexural strength increased gradually with the increase of BF volume dosage. When the dosage of FA was 0% and the BF volume dosage admixture was increased from 0 to 0.3%, the flexural strength of concrete was increased by 25.90%.

From the above results, a moderate amount of nano-TiC, BF, and FA could improve the flexural strength of concrete. The main reason was that the appropriate amount of BF formed a mesh structure inside the concrete matrix, which shared the external load with the matrix through interfacial bonding^47^. The FA improved the fluidity of concrete leading to a more uniform dispersion of fibers in concrete^48^. The close-packed structure formed by nano-TiC with BF and FA in concrete further enhances the flexural strength of concrete. The optimal mix ratio for flexural strength was 1.34% for nano-TiC, 0.24% by volume for BF, and 6.89% for FA.

### The design optimization and result analysis of modified concrete

The maximum values of concrete compressive, split tensile, and flexural strengths were selected based on the parameter ranges in Table 7. Model synthesis optimization was carried out within the range of factor levels. The optimum admixture of nano-TiC, BF, and FA in concrete was obtained as 0.88%, 0.24%, and 5.49%. To further determine the difference between the predicted and actual values of the RSM optimization results. Three sets of parallel tests were carried out under the same conditions with the optimal dosage and the actual measurements were compared with the predicted values (Table 8). The maximum error between the predicted and the actual values of the compressive strength of TBF concrete was 6.09% relative error. The maximum error between the predicted and actual values of split tensile strength was 7.14%. The maximum error in flexural strength is 8.45%. This indicates that the RSM model had better accuracy and could better reflect the actual measurement results of concrete. Table 9 shows the comparison between the predicted values of TBF0 without the addition of nano-TiC, BF, and FA and the optimized TBF concrete, and the mechanical properties of the concrete were obviously improved.

**Table 7:** Parameter intervals and optimization of TBF concrete.

Form	Goal	Lower limit	Upper limit
*A:* nano-TiC	Is in rage	0	2.5
*B:* BF	Is in rage	0	0.3
*C:* FA	Is in rage	0	15
Compressive strength	Maximize	32.5 MPa	49.5 MPa
Splitting tensile strength	Maximize	4.1 MPa	5.6 MPa
Flexural strength	Maximize	6.32 MPa	8.25 MPa

**Table 8:** The RSM response design optimization results.

Test group	Predicted value/MPa			Actual value/%			Inaccuracy/%		
	Compressive	Splitting tensile	Flexural	Compressive	Splitting tensile	Flexural	Compressive	Splitting tensile	Flexural
1	45.3	5.2	8.28	42.7	5.1	7.81	6.09	1.96	6.02
2				46.6	5.6	8.35	2.80	7.14	0.84
3				45.2	5.5	7.58	0.22	5.45	8.45

**Table 9:** The TBF0 and predicted value mechanical strength.

Typology	Compressive/MPa	Splitting tensile/MPa	Flexural/MPa	Promotion rate/%		
				Compressive	Splitting tensile	Flexural
TBF0	37.5	4.5	6.89	–	–	–
Projected value	45.3	5.2	8.28	20.80	15.56	20.17

### The microscopic morphology of modified concrete

**Figure 7:**
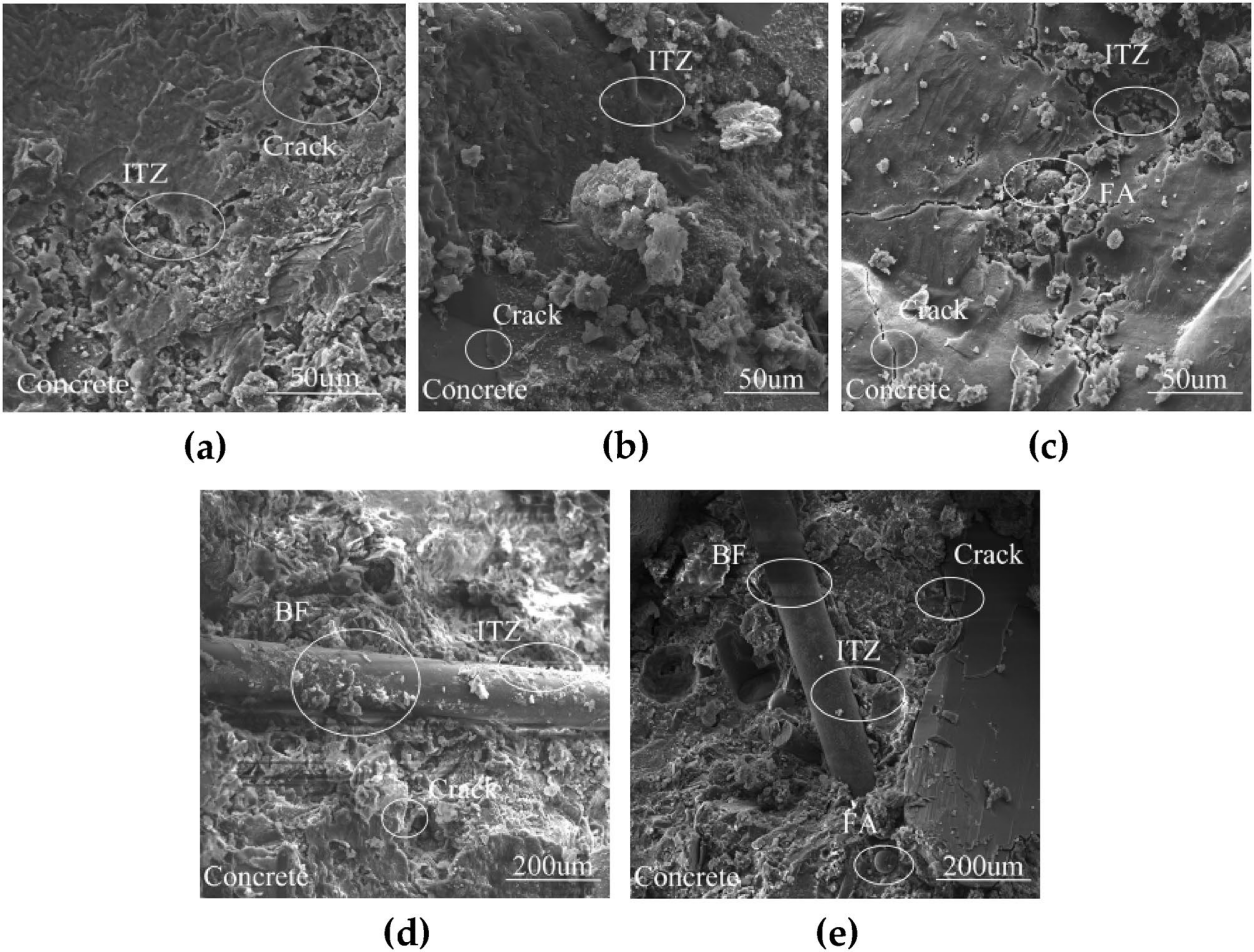
SEM picture of concrete: (**a**) TBF0, (**b**) TBF8, (**c**) TBF16, (**d**) TBF3, (**e**) TBF5.

Figure 7a shows the SEM image of TBF0 after the pressurized test. The concrete had obvious cracks after crushing, which was mainly a phenomenon caused by stress concentration in the internal weak areas after the concrete was subjected to the applied load. Moreover, some porosity appeared in some areas of the image.

Figure 7b shows the SEM image of single-mixed nano-TiC concrete (TBF8) after a pressurized test. Compared with the TBF0, the interfacial transition zone (ITZ) became less, the pore volume decreased, and the whole became dense. And "spherical" particles with smooth surfaces appeared in Fig. 7c, which was mainly due to FA which was not involved in hydration. Moreover, some of the spherical particles had hydrates "attached" to their surface, which was mainly due to the participation of FA in the secondary hydration, a phenomenon that contributed to the improvement of the concrete strength in the later stages of the process^49^.

Figure 7d shows the SEM images of single-mixed BF concrete (TBF3) after crushing by the testing apparatus. There was an ITZ between BF and concrete aggregate, which was mainly due to the interfacial bonding between the fibers and concrete aggregate. In addition, the cracks and ITZs in the concrete with single-mixed BF were weaker than those in TBF0. The main reason why the concrete was able to maintain the integrity of the concrete specimens better after reaching the load-bearing capacity limit.

Figure 7e shows the SEM image of mixed nano-TiC, FA, and BF concrete (TBF5) after crushing. There were a few cracks in the figure and the cement was well cemented. It may be because the appropriate amount of BF, FA, and nano-TiC improved the densification of the concrete and optimized the structure of the concrete matrix. Moreover, the tensile stress provided by the fibers when they were pulled out of the concrete under external loads further delayed and reduced the crack generation.

## Conclusions

In this study, RSM was used to optimize the concrete with nano-TiC, BF, and FA admixtures. The effects of the three admixtures on the collapse, compressive, splitting tensile, and flexural resistance of concrete were investigated. The concrete conclusions were as follows:

- The incorporation of nano-TiC and BF in concrete decreased the concrete's degree of collapse. Whereas, the incorporation of FA improved the collapse of concrete.
- When nano-TiC and FA were mixed in concrete alone. Under the ultimate load, some of the cement paste on the surface of the test block fell off and could not maintain its integrity. Whereas, when appropriate amounts of nano-TiC, FA, and BF were mixed in concrete. Few or no shedding on the surface of the specimen could maintain its integrity. Their SEM pictures were also relatively dense.
- The mechanical properties of concrete were affected by the admixture of nano-TiC, FA, and BF. The effect of nano-TiC on the compressive strength of concrete was remarkable, while the effect of BF and FA on the compressive strength of concrete was very substantial. The effect of BF and FA on the split tensile and flexural strength of concrete was highly significant. The effect of the interaction of nano-TiC and BF on the compressive strength of concrete was significant. The effect of the interaction of FA and BF on the split tensile and flexural strength of concrete was highly significant.
- By establishing a thirst function optimization model. The optimum mix ratios for compressive, split tensile, and flexural strength of concrete were obtained. The dosages of nano-TiC, BF, and FA were 0.88%, 0.24%, and 5.49%, respectively. The accuracy of the optimization results was also verified by the same test conditions, and the fit of the model was higher. It was of practical significance to study the application of concrete in construction engineering.

In conclusion, the mechanical properties of TBF concrete were tested and the microstructure was analyzed in this study. The relationship between nano-TiC, BF, FA, and concrete was determined. In the future, the concrete can also be combined with numerical simulation and microanalysis. The effects of nano-TiC, BF, and FA on the freeze–thaw capacity of concrete were studied in depth.

## Data Availability

The available figures presented in this research are obtainable upon contact with the corresponding authors. Not being open to public scientific, these digital will be part of our continuing investigation.
